# Development and validation of the Continuous Traumatic Stress Response scale (CTSR) among adults exposed to ongoing security threats

**DOI:** 10.1371/journal.pone.0251724

**Published:** 2021-05-27

**Authors:** Aviva Goral, Paula Feder-Bubis, Mooli Lahad, Sandro Galea, Norm O’Rourke, Limor Aharonson-Daniel

**Affiliations:** 1 School of Public Health, Faculty of Health Sciences, Ben-Gurion University of the Negev, Be’er Sheva, Israel; 2 PREPARED Center for Emergency Response Research, Ben-Gurion University of the Negev, Be’er Sheva, Israel; 3 Department of Health Systems Management, School of Public Health, Faculty of Health Sciences and Guilford Glazer Faculty of Business and Management, Ben-Gurion University of the Negev, Be’er Sheva, Israel; 4 Department of Psychology, Tel-Hai College, Upper Galilee, Israel; 5 The Community Stress Prevention Centre (CSPC), Kiryat-Shmona, Israel; 6 School of Public Health, Boston University, Boston, MA, United States of America; University of São Paulo, BRAZIL

## Abstract

**Background:**

Current diagnostic criteria for posttraumatic stress disorder (PTSD) do not include symptoms resulting from exposure to continuous or ongoing traumatic stress. Thus existing assessment tools do not fully capture stress symptoms associated with exposure to threats that extend over months or years. To address this void, we enumerated the symptoms associate with ongoing exposure to stress including those that are distinct from existing PTSD diagnostic criteria.

**Objectives:**

To develop the Continuous Traumatic Stress Response Scale (CTSR) and assess its psychometric properties.

**Method:**

We sampled 313 adults exposed and unexposed to ongoing security threat between December 2016 and February 2017. Respondents lived in communities bordering the Gaza Strip in southern Israel where they are exposed to frequent rocket attacks, requiring they locate and find shelter in 30 seconds or less. We assessed the concurrent validity of CTSR relative to the Posttraumatic Diagnostic Scale (PDS).

**Results:**

On the basis of exploratory factor analysis (EFA), we retained 11 of 25 items measuring three distinct factors: exhaustion/detachment, rage/betrayal, and fear/helplessness. We found moderate concurrence between the scales; that is, the CTSR appears to measure a construct related to, but distinct from PTSD. This conclusion is supported by confirmatory factor analysis (CFA) indicating that each factor significantly contributes to measurement of a higher-order, continuous traumatic stress latent construct.

**Conclusions:**

These results support the psychometric properties of CTSR. Future research is required to confirm these findings in other countries and cultures and among individuals exposed to other forms of continuous traumatic stress.

## Introduction

There is growing recognition that the traumatic effects of ongoing exposure to traumatic stress extend beyond current diagnostic criteria for posttraumatic stress disorder [PTSD; 1–4]. Studies show that the responses to ongoing exposure to threat are wider and more intensive than those associated with a single traumatic exposure [1, 2, 4–8]. Additional responses arising from multiple or prolonged exposure to threat may include cognitive, behavioral and emotional effects, including anxiety, depressive, somatization, heightened risk perception, constant concern for the future, mental exhaustion and change in sense of self [7, 9–11]. Some of these are not listed or described in DSM-5 criteria for PTSD [6, 9, 10].

Ongoing traumatic stress response [OTSR; 1] and continuous traumatic stress [CTS; 12] are the two most common terms proposed to distinguish and define the effects of ongoing exposure to threat. Both focus on present and future trauma exposure, rather than on a finite, past traumatic event; and both challenge the pathological characteristic of PTSD in the context of ongoing exposure to traumatic stress. According to Eagle and Kaminer [12] "CTS captures a domain of traumatic stress experience not adequately formulated in the existing repertoire of traumatic stress responses but which characterizes the lived experience of many individuals and communities around the globe". Furthermore, a review study of the psychological aspects of the Israeli-Palestinian conflict [7], show that nearly all studies assessing functional impairement (7 out of 8) found that greater exposure to political conflict and violence is associated with more severe functional impairment. While the traumatic effects of ongoing exposure to security threats may be viewed as normal, adaptive responses to abnormal, realistically threatening conditions, they can also have detrimental effects, requiring ongoing regulation and management. Taken together, these findings suggest that the current conceptualization of PTSD may have limited applicability to ongoing threat exposure, underscoring the need for a separate diagnostic construct that differentiates between the effects of ongoing exposure to threat from those observed in a single, past traumatic event.

Both OTSR and CTS emphasize anticipatory anxiety and its impact. Pat-Horenczyk [13], used the term ‘near miss exposure’ to capture the experience of missing the traumatic event, by chance, often by just moments, and the ability to envision *what if* (i.e., the realistic possibility of being hurt in the future). Indeed, emerging research suggests that *anticipated trauma* underlies the anxiety associated with ongoing exposure to security threat [1, 14] and may be central to the development of posttraumatic psychopathology [15–18].

Other stress symptoms may stem from the cumulative threat exposure, consistent with the construct of *allostatic load* [19]. Exposure to repeated or persistent stress creates ’wear and tear’ on the mind and body, which in turn, impedes coping and accelerate disease progression. Respite and regulation of physiologic systems is integral to coping with adversity, and is associated with physical and mental health outcomes [20–22]. Previous studies indicate that ongoing exposure to threat is associated with mental exhaustion [9] and increased morbidity [22, 23].

Most research examining the consequences of ongoing exposure to security threats has focused on narrower personal resources and symptom clusters. These included sleep problems [24], resilience [25], adult attachment [26], satisfaction with life, prejudicial attitudes [27], sense of safety, optimism, help-seeking [28], anxiety, depression, posttraumatic recovery [29, 30], and physical health [22, 31]. Itzhaky et al. [10], identified distinct psychiatric symptom *profiles* associated with CTS exposure, in addition to PTSD diagnostic criteria. To the best of our knowledge, we are the first to systematically identify and measure the phenomena associated with exposure to ongoing security threats.

Exposure to ongoing risk of harm is a global phenomenon, existing wherever people are exposed to ongoing terror, rampant crime and civil war. Thorough understanding and effective measurement of the effects of persistent or ongoing trauma is necessary to identify and treat those affected. However, no scales currently exist to assess the broader effects of exposure to ongoing traumatic stress aside from PTSD measures. This paucity of screening tools combined with limited evidence-based treatment [32], limit both the assessment and clinical practice, and may lead to misdiagnoses and missed and/or ineffective treatment. The current study was conducted to fill this gap, as we developed a new comprehensive tool to measure the symptoms associated with exposure to ongoing security threats. In this manuscript, we describe the tool building process. We describe the procedures followed for scale construction and validation and the results supporting the new scale.

## Methods

### Ongoing security threat: Exposed vs. unexposed participants

Among the communities most affected by the ongoing Israeli-Palestinian conflict are those bordering the Gaza Strip in southern Israel who have been exposed to persistent ’low intensity’ war-like conditions since 2001 (e.g., frequent rocket shelling). This low-level exposure is punctuated by periods of high intensity conflict. During ‘Operation Protective Edge’ in the summer of 2014, roughly 3,400 projectiles were fired from the Gaza Strip, 4,600 sirens were heard throughout the country (average 92 per day). Most sirens were in communities closest to Gaza where residents have only 15 seconds to secure shelter before rockets land. Residents living in border communities are also threatened by infiltration via underground tunnels and incendiary balloons targeting children. Communities further from the Gaza Strip (e.g., Tel Aviv) are protected by the Iron Dome anti-Missile Defense System that intercepts and destroys rockets with longer trajectories. All new homes and buildings in Israel need to have a secure room with a reinforced door, others have outdoor protective structures, or rely on communal shelters in residential neighborhoods. Yet some residents, even in communities bordering the Gaza Strip, have no access to secure shelter; for others, reaching a shelter is physically challenging or even impossible (e.g., elderly, disabled) exposing them to greater risk of injury.

We distinguish communities within 20 km of the Gaza Strip with documented high exposure to rocket/missile attacks (Western Negev region, please see S1 Table), as *exposed to continuous security related traumatic stress* (CTS), whereas those at greater distance are *unexposed to continuous security related stress* (non-CTS; relatively low exposure). For this study, we assumed that adults living in high versus low exposure communities would report more symptoms suggestive of continuous traumatic stress (e.g., short vs. longer siren warning zones). This result would support the construct validity of responses.

### Recruitment and data collection

We hired iPanel, an online survey agency to recruit participants. iPanel is one of the largest survey panels in Israel, widely used by research institutes and market research companies as an online platform for a wide variety of information collection services. iPanel adheres to the stringent standards of the European Society for Opinion and Marketing Research (ESOMAR). Study participants were adults, stratified by age and gender to match the socio-demographics features of the Western Negev according to census data [33]. High and low exposure communities were matched by community type (urban/rural). Participants were also recruited by the directors of resilience centers in communities bordering the Gaza Strip. Finally, we posted recruitment notices on social media platforms (e.g., Facebook, WhatsApp). Participation was voluntary given the inclusion criteria (age 18+ years, one questionnaire per household) were met. Survey participants were informed that by agreeing to participate in the study they were providing their written informed consent. Data protection was observed throughout the study.

Data were collected online providing an initial sample of 558 respondents between December 2016 and February 2017; 313 (56.1%) were retained for analyses whereas 245 were excluded due to incomplete data (of these, 198 were excluded due to fully skipped CTSR questions, 47 were excluded due to wrong answers to attention checks embedded within CTSR items). Participants providing complete and incomplete responses were demographically similar (i.e., age, gender, family status, income level, education) suggesting that completeness did not confound study responses. This study received ethical committee approval from the Faculty of Health Sciences at Ben-Gurion University of the Negev, Be’er Sheva, Israel.

### Procedures

#### Scale development

Scale development and initial validation consisted of four phases intended to fully elicit the symptoms most salient to ongoing traumatic stress and devise a self-report scale to measure this construct and its component factors. These phases included; conceptualization and item generation; breadth of measurement and content validity; development and assessment of psychometric properties (e.g., internal consistency); and initial validation of the new scale [34].

We first conducted an extensive literature review to inform in-depth interviews with key informants, two focus groups sessions, and two academic discussions and expert workshops. Six interviews were conducted with four psychologists and two social workers all experienced in treating trauma victims. The first focus group consisted of 11 therapists (three psychologists, one psychotherapist, four social workers, one social worker/psychotherapist and two drama therapists). The second focus group consisted of seven therapists (five psychologists, one social worker, and one psychotherapist/ social worker). All focus group participants were experienced in treating trauma victims and individuals exposed to ongoing security threat, all were trained in various trauma treatment techniques. Via a survey of therapists, we set out to define the symptoms most salient to ongoing exposure to security related traumatic stress [9].

This multi-prong approach provided us with multiple viewpoints and perspectives to thoroughly understand and define the construct. The item generation process allowed us to compile an initial pool of 74 items from the literature, key informants, and existing PTSD scales. Based on expert opinion [35], 25 items were identified as most relevant responses to ongoing traumatic stress. Some are distinct from PTSD diagnostic criteria (e.g., “I feel that my life is in danger”; “I feel mentally exhausted”), while other items reflect PTSD diagnostic criteria (i.e., “I find it hard to trust the people around me”), but not explicitly measured by current assessment tools. Other items correspond to Disorders of Extreme Stress (DESNOS) diagnostic criteria, designed to assess complex PTSD, i.e., trauma that is prolonged in duration, occurring early in life and is of interpersonal nature such as childhood sexual abuse and neglect [36, 37]. DESNOS diagnosis was found to be more common in individuals exposed to interpersonal trauma of early onset and of lasting duration than in other types of traumatic experiences [37]. DESNOS criteria are thus unsuitable for assessing traumatic stress in ongoing exposure to security threat.

### Measures

#### Prospective item pool

After initial analyses, the 25 items most relevant to ongoing traumatic stress were retained. Items were administered in Hebrew, participants were asked to report the occurrence and severity over the last month with responses ranging from *not at all* (0) to *severe* (3). A total scale score was calculated as the sum of all items that remained following the exploratory factor analysis process (described below). Similarly, each factors’ score was calculated as the sum of all items loaded onto the same factor. Two final questions ask respondents to report ’the level of distress’ and ’the level of interference with everyday functioning’ on a 5-point scale ranging from *no distress / functional impairment* (1) to *severe distress / functional impairment* (5). Distress and functional impairment levels were further categorized as low (2 or less) or high (more than 2).

#### History of trauma and posttraumatic stress

Participants reported exposure to trauma (yes/no) or other adverse events. Lifetime exposure to trauma was defined as a ’threat to your life or the life of someone close to you, to your physical or mental integrity, including serious injury, the threat of death to you or someone close to you’. Additional trauma related information included the type of trauma, which was verified against the list of potential traumatic events specified in the Life Events Checklist for DSM-5 [LEC-5; 38], who was exposed (e.g., self, first degree relative, other relative / friend), the extent of injury, and time since the traumatic event. Participants were classified as having a direct traumatic event (corresponding to DSM-5 criteria A), if they reported being present, injured or witnessed, or a close friend / a family member was injured or died due to a traumatic event.

Posttraumatic stress symptoms were assessed with the Posttraumatic Diagnostic Scale [PDS-5; 39] which corresponds to DSM-5 diagnostic criteria and has good internal consistency (α = .95). Participants indicated the occurrence and severity of 20 PTSD symptoms over the past month along a four point Likert scale (*0 = not at all*, to *3 = severe*). Responses of 2 (very much) or higher were considered clinically significant and were counted as endorsed symptoms. In accord with DSM 5, those who endorsed 1+ intrusion symptom, 1+ avoidance symptom, 2+ negative changes in cognition / mood, and 2+ changes in arousal / reactivity were assumed to meet PTSD symptoms criteria (regardless of exposure to trauma). Internal consistency for the full PDS scale in the current study was high (α = .93).

#### Perceived likelihood and fear of terror

Participants were asked to report the perceived likelihood and associated fear of injury related to various acts of terror (e.g., missile attack near home, terrorist attack near home, attack tunnel detected nearby, shooting). Responses to items range from *no fear/threat* (1) to *great fear /threat* (5). Total fear and likelihood scores were calculated as the sum of items for each scale. Internal consistency ranged from α = .75 for the likelihood subscale to α = .90 for the fear subscale.

#### Sociodemographic data and emergency preparedness

Participants provided sociodemographic information (age, gender, birth country, family status, household income, education, religiosity, distance from the Gaza border based on participants’ post code, duration at current address), as well as information related to their functional abilities (being physically disabled, providing care to others such as a small child, an elderly or a disabled person) and shelter availability.

### Analytic procedures

Participants were randomly divided in two groups: one for exploratory factor analysis (EFA; *n* = 113) and one for confirmatory factor analysis (CFA; *n* = 200). Using the formulae for SAS based on the RMSEA statistic (df = 35), we estimated statistical power for the CFA model at .64, sufficient to detect medium to large effects. These numbers are not high, but meet minimum sample size requirements for both procedures [40, 41]. The two samples do not statistically differ in terms of age, gender, family status, income level and education level. EFA was first performed on the pool of prospective items to achieve a working version of the scale. Principal axis factoring was used with varimax rotation consistent with other scale construction research [e.g., 42, 43]. EFA was performed using SPSS 21.

Confirmatory factor analysis (CFA) was next performed to confirm EFA results and to ascertain if first-order factors map onto a higher-order latent construct. Three goodness-of-fit-indices are reported to assess the overall fit of path models: an incremental, an absolute, and a parsimonious fit index. The Comparative Fit Index (CFI) is an incremental index representing the extent to which a hypothesized model is a better fit to data than the null model. Coefficient values greater than .94 for the CFI indicate good model fit. The Standardized Root Mean Square Residual (SRMR) is an absolute index which represents the standardized difference between observed and predicted correlations within a hypothesized model [44]. Finally, the Root Mean Square Error of Approximation (RMSEA) is a parsimony index, which represents the extent to which a hypothesized model fits data relative to the general population. Coefficient values less than .055 for the SRMR and RMSEA suggest good model fit [45]. AMOS 22.0 was used to perform CFA.

With a final version of the scale following CFA analysis, we first computed descriptive statistics for the full scale and for each factor (i.e., internal consistency). We then examined the degree to which the construct measured in the new scale is related to (or differs from) the PTSD scale (i.e., concurrent validity).

We used the median value of 3 as a cutoff point to identify individuals with higher or more severe CTSR scores. Using this cutoff point, we then assessed the scale’s construct validity vis-à-vis socio-demographic variables. We first assessed CTSR score predictors using a hierarchical multivariate regression model adjusting for sample differences and other covariates (significant at p<0.2 in univariate models). In the first step, we entered sociodemographic variables (age, gender, family status, religiosity, income level, community type), past exposure to trauma, access to secure shelter and physical disability. In order to assess the unique contribution of the level of perceived odds and exposure to ongoing security threat, they were entered separately in steps two (odds of event) and three (CTS) of the model. The interaction between ongoing exposure to security threat and level of perceived odds of future traumatic events was entered in step 4. Finally, we assessed the association between the level of distress / functional impairment, distance from the Gaza border and CTSR scores. Statistical analyses were conducted using SPSS 21.

## Results

### Research sample

Our sample consisted of 313 adults, 138 (44.1%) exposed to ongoing security threat (mean distance from the Gaza border = 7.73 km, standard deviation, *SD* = 4.29 km) and 175 (55.9%) unexposed (mean distance from Gaza = 66.58 km, *SD* = 17.65 km). The mean age was 41.1 years (*SD* = 13.05 years), most were female (n = 190, 61.1%). About 35.0% indicated prior exposure to trauma (n = 109) and 93 (29.7%) appear to meet DSM-5 criteria for post-traumatic stress symptoms (PTS; regardless of exposure to trauma). The CTS and non-CTS participants differed in age, gender, family status, religiosity level, income level, birth country, community type, and access to shelter (Table 1). Significant differences were also found in mean level of perceived fear (*t* = 6.66, p<0.001) and mean perceived likelihood of future terror related events (*t* = 8.01, p<0.001). Please see S2 Table for more statistical details.

**Table 1:** Descriptive statistics by exposure to continuous security threat.

Demographics characteristic	All	CTS	Non-CTS (N = 175)	P
	(N = 313)	(N = 138)		
Age, mean (SD)	41.1(13.0)	42.1(14.3)	39.7 (12.1)	0.010
Female	189(60.4%)	96(69.6%)	93(53.1%)	0.001
Married or cohabitating	231(73.8%)	93(67.4%)	138(78.8%)	0.045
Above average income	104(33.3%)	36(26.1%)	68(38.8%)	0.024
Non-religious	184(59.1%)	92(66.7%)	92(52.6%)	0.006
Academic education	215(68.7%)	90(65.2%)	125(71.4%)	0.220
Israeli*	257(82.1%)	103(74.6%)	154(88.0%)	0.001
Urban	152(48.6%)	52(37.7%)	100(57.1%)	0.001
**Trauma related characteristics**				
Prior exposure to trauma	109(34.8%)	44(31.9%)	65(37.1%)	0.350
PTSD symptoms**	93(29.7%)	37(26.8%)	56(32.0%)	0.200
CTSR symptoms	140(45.2)	67(48.6%)	73(42.4%)	0.280
Fear of events, mean (SD)	2.58(1.16)	3.04(1.15)	2.21(1.02)	<0.001
Likelihood of events, mean (SD)	2.49(0.77)	2.85(0.75)	2.21(0.66)	<0.001

*Born in Israel or immigrated before 1990

**Symptoms only, regardless of ’exposure to a traumatic event’ as defined by Criteria A

### Exploratory factor analysis

The Kaiser-Myer Olkin value for the 25 item pool model was high (KMO = 0.88) indicating good internal consistency, sufficient for EFA. Eleven of 25 items were retained following EFA. Based on eigenvalues, scree test shows a discernible flattening of the plot after the third factor, suggesting a three-factor structure. Each factor is measured by 3+ items as recommended for scale construction [45]. These three factors account for 61.62% of observed variance. Key EFA components are presented in S3 Table (Eigenvalues) and S1 Fig (Scree Plot).

### Confirmatory factor analysis

**Fig 1:**
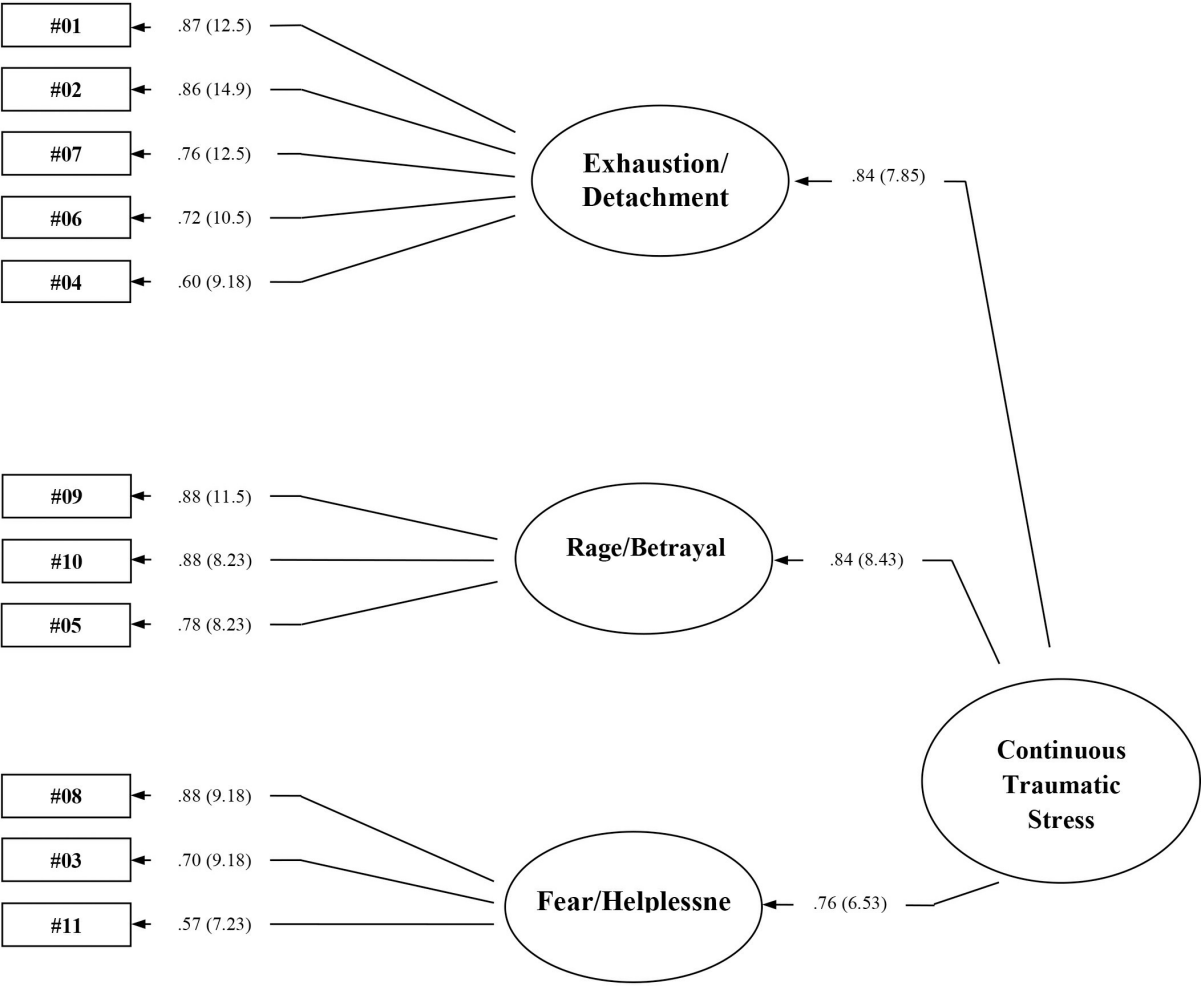
A 3-factor, higher-order model for continuous traumatic stress. Standardized estimates for items and factors are presented. Parenthetical values represent significance estimates (all greater than 1.96). CTS items include: items 1 (I feel unmotivated), 2 (I feel mentally exhausted), 4 (I feel that my life has no meaning), 6 (I find it hard to trust the people around me), and 7 (I feel that no one understands me) in the exhaustion and detachment factor; items 5 (I have difficulty controlling my emotions), 9 (I have episodes of rage), and 10 (I feel betrayed) in the rage and betrayal factor; and items 3 (I feel that my life is in danger), 8 (I have intense feelings of fear or horror), and 11 (I feel that I cannot protect the people who depend on me), in the fear and helplessness factor.

We next performed CFA to confirm the measurement properties of this prospective 3-factor model. As hypothesized, each item significantly measured its respective factor and each of these three factors significantly measured a higher-order latent construct. Statistics indicated excellent goodness of fit between the model and data after correction for correlated error for 8 of 104 possible error terms, χ^2^ = 49.82, *df* = 35, *p* = .05. More precisely, the comparative fit index is in ideal parameters (i.e., CFI>.94, CFI = .99), as is for the standardized root mean residual (SRMR < .055; SRMR = .044), and the root mean square error of approximation (RMSEA < .055; RMSEA = .046). The full 90% confidence interval for the RMSEA is in acceptable parameters (.001<RMSEA CL_90_ < .073). Statistical power for this CFA model was estimated at .64 (n = 200, df = 35), sufficient to detect medium to large effects [45] (Fig 1).

### Continuous exposure to threat scale and factor structure

We have provisionally labeled the factors: 1. *Exhaustion / emotional detachment* (5 items; e.g., I feel mentally exhausted); *2*. *Rage / betrayal*, (3 items; e.g., I feel betrayed); and 3. *Fear / helplessness* (3 items; e.g., I feel I cannot protect those who depend on me). The internal consistency was α = .90 for the total scale, α = 0.86 for *Exhaustion / emotional detachment*, α = .82 for *Rage / betrayal*, and α = .74 for *Fear / helplessness*. Mild to moderate correlations were found between the three factors. Correlation coefficient values ranged between r = .511 (exhaustion/detachment and fear/ helplessness) and r = .649 (exhaustion/detachment and rage/betrayal; p < .001 for all).

### Concurrent validity

Concurrent validity was assessed by examining the correlations between the PTSD and the new scales. Responses to both scales were strongly correlated (r = 0.720, p < .001) with smaller coefficients between the PTSD scale and each of the separate CTSR factors (r = .60 for fear / helplessness, and r = .62 for both exhaustion / emotional detachment and rage / betrayal factors; p < .001 for all). Less than 40% of the variance in stress symptoms is accounted for by any single correlation between the PTSD scale and each factor (35% to 39%), demonstrating that the two scales measure related but distinct constructs.

### Construct validity

Descriptive analyses showed that responses to the new scale are associated with gender, family status, income level, past exposure to trauma, perceived fear, perceived likelihood of future terror (Table 2), and age (*t* = 5.29, *p*<0.001). In order to determine if perceived fear or likelihood of future traumatic events moderate the relationship between exposure to ongoing security threat and scale scores, we computed two interaction terms between exposure to security threat (CTS/non-CTS), level of perceived fear, and likelihood of future trauma (both were person mean‐centered prior to computing interactions). Both interaction terms were statistically significant.

**Table 2:** High CTSR* scores prevalence, by sample characteristics (n = 311).

Parameter		CTSR		Χ^2^(df),P
		N	%	
**Gender**	Male	96	50.8%	6.0(1),0.01
	Female	42	35.9%	
**Religiosity level**	Secular	75	41.2%	3.2(2),0.24
	Traditional	30	53.6%	
	Religious	34	49.3%	
**Family status**	Married or cohabitating	95	41.1%	9.6(2),0.007
	Single	33	64.7%	
	Divorced or widow	11	40.7%	
**Income level**	Lower than average	57	53.8%	8.1(2),0.02
	Like average	47	47.5%	
	Higher than average	36	34.6%	
**Community type**	Rural	75	50.3%	2.24(1),0.09
	Urban	64	40.3%	
**Exposure to trauma**	No	83	41.1%	4.2(1),0.04
	Yes	58	53.2%	
**Fear of future events**	≤10	59	33.3%	23.4(1),<0.001
	>10	80	61.1%	
**Likelihood of future events**	≤12	67	37.2%	11.4(1),<0.001
	>12	74	56.5%	
**Physical handicap**	No	118	43.5%	2.3(1),0.13
	Yes	22	56.4%	
**Ongoing exposure to security threat**	No	74	42.8%	1.1(1),0.28
	Yes	67	48.6%	

*The median value of 3 was used as a cutoff point to identify individuals with high CTSR scores

Results of the multivariate regression analysis showed that after adjusting for sample differences, new scale scores were significantly predicted by age (AOR 0.96; 95% CI 0.94–0.99, p = 0.003, for a one year increase in age), female gender (AOR 1.81; 95% CI 1.05–3.14, p = 0.03) and perceived likelihood of future terror (AOR 1.11; 95% CI 1.03–1.20, p = 0.007). The interactions between ongoing exposure and perceived fear / likelihood of future terror were non-significant and were thus removed from the model. Please see S4 Table for results of the final adjusted regression model.

#### Association between new scale scores and the level of distress / functional impairment and distance from the Gaza border

The association between new scale scores and the level of distress / functional impairment and distance from the Gaza border were assessed using Spearman rank-order correlations. New scale scores were strongly correlated with both the level of distress (r = .726, p<0.001) and functional impairment (r = .686, p<0.001). Please see S2 and S3 Figs for scatterplots. Furthermore, the vast majority (94.2%) of participants categorized as having high CTSR scores did not meet PTSD criteria *in accord with DSM 5* and only 62.2% were endorsed as having PTSD symptoms (in accord with DSM 5 regardless of exposure to trauma). New scale scores were significantly correlated with the distance from the Gaza border among CTS participants (r = .265, p = 0.002) but not among non-CTS participants (r = .014, p = 0.855. (Please see S4 Fig for scatterplots).

## Discussion

This study was motivated by the clinical observation that the effects of ongoing trauma extend beyond those assessed by PTSD criteria [3, 10]. PTSD criteria in DSM-5 were designed for assessing posttraumatic stress in single-event traumas, limiting its’ diagnostic capabilities in multiple traumatic events in general, and in ongoing exposure to security threat in particular [46]. Criterion A requires a specific traumatic exposure to serve as the basis for PTSD diagnosis, and in the case of multiple traumatic exposures, symptoms are assessed with regards to the worst traumatic event. However, in ongoing traumatic exposures, stress symptoms relate to both the past and potential future traumatic events. In that respect, Criterion A does not take into account the effect of fear from potential future traumatic events or the cumulative effect of ongoing exposure to threat [1, 8]. The traumatic stress picture associated with ongoing exposure to security threat is thus not the same as the DSM-5 assessed PTSD. Furthermore, several fundamental aspects such as avoidance behavior that is connected to real life survival (i.e. going out in times of tension and risk of being injured) or sleep disturbance in times of ongoing midnight parades and shelling are a reasonable reaction to a threat. Another finding that demonstrates the gap between response to ongoing threat and the known PTSD is that once a resident of the region leaves the affected area, symptoms subside dramatically.

Commonly used assessment tools may thus be insufficient to measure symptoms where threats vary in nature and intensity yet persist over time (e.g., sporadic points of military conflict). Following procedures for scale construction and validation, we designed a scale to measure traumatic stress resulting from ongoing exposure to security threat. Based upon our analyses, we propose the Continuous Traumatic Stress Response Scale (CTSR; 11 items) measuring three factors labeled: *Exhaustion/ detachment*; *rage/betrayal*; *fear and helplessness* (please see S1 File for items included and un-included in the final CTSR scale).

CFA results indicate that these three first-order factors map onto a higher-order continuous traumatic stress latent construct. Different from the structure and content of PTSD symptoms clusters, these factors suggest that there are distinct clinical features resulting from acute versus persistent, seemingly unending threats. This is consistent with the wear-and-tear hypothesis and allostatic load [19]. In other words, repeated attacks and new forms of terror reinforce the fear that the threat will never end. Psychological well-being erodes over time with seemingly inadequate response by authorities fostering rage, exhaustion and helplessness over time.

Allostasis and allostatic load can serve as organizing concepts for understanding both the short-term adaptation to acute stressors and the pathophysiology that is often associated with chronic anxiety-related disorders [47] as the one we address in this manuscript. In this 2003 article, McEwen refers to the situation in which the mediators of allostasis are dysregulated over many months and years. Similarly, we suggest that CTS can be seen as a focused measure of long-term exposure to threat resulting in exhaustion/detachment; rage/betrayal; and fear/helplessness, which are not alien to PTSD but are specific to the case which we studied. The CTSR may thus offer clinicians a more specific understanding, as well as a reference to the manifestation of PTSD in such circumstances.

The final version of the CTSR scale consists items distinct from PTSD diagnostic criteria (i.e., mental exhaustion, reduced sense of safety, inability to protect the people who depend on me), or may be understood as extensions of PTSD symptom clusters (i.e., feeling of betrayal, feeling misunderstood, difficulties in emotion regulation). Nevertheless, none is *specifically* defined by existing diagnostic criteria nor in therapeutic protocols for treating those exposed to ongoing threat.

Among CTSR items, reduced sense of safety [30, 48, 49], distrust [31], and mental exhaustion [31, 50] each emerged as associated with ongoing exposure to stressors. Other items including social withdrawal, feelings of emptiness, hopelessness, estrangement, and feeling of being constantly threatened, are associated with change in sense of self which is also a consequence of ongoing exposure to trauma [51–53].

Our results show that perceived likelihood of injury due to various terror related traumatic events was a strong predictor of severe CTSR scores. These findings imply that it is not exposure to ongoing threats per se, but rather the level of perceived threat (i.e., perceived fear and perceived likelihood of injury), that accounts for the difference in CTSR stress symptoms prevalence and severity. Compared with distant communities, border adjacent communities are much more vulnerable to rockets or tunnels infiltration creating an atmosphere of tension and fear. Indeed, both fear level and likelihood of injury from terror-related events were significantly higher among CTS participants compared to non-CTS participants, as was also reflected by the items included in factor 3 (*fear and helplessness*). It has been suggested that when exposed to ongoing threats, individuals in CTS communities experience collective, increased fear and anxiety which has the potential to further aggravate stress symptoms and traumatic stress symptoms risk [26].

In addition, the association between CTSR severity and past exposure to trauma was non-significant, implying that unlike PTSD symptoms, CTS symptoms severity is not a function of prior exposure to trauma and should not be required as a prerequisite for diagnosis and further treatment. CTSR should thus be addressed in the therapeutic process regardless of prior exposure to trauma, terror or non-terror related. These findings provide support for the argument concerning the applicability of DSM-5 criteria A when assessing stress symptoms among individuals exposed to ongoing security threat [3]. Finally, our study shows that CTSR is associated with high distress and functional impairment levels further strengthening the need for a more nuanced measurement tool.

### Study limitations

We recruited a convenience sample reflecting the sociodemographic composition of the Western Negev region, yet we do not know what percentage of prospective participants declined to take part in this study. This limits our ability to provide population norms at this juncture. Nor can we generalize findings to other regions, conflicts or language groups.

Further research should be conducted with larger samples, in English and other languages and with other populations exposed to ongoing conflict or persistent civil war (e.g., Syria). This will enable cross-cultural research identifying the similarities and differences between conflict zones or language groups, and will further promote the generalizability and validity of the CTSR scale.

## Conclusions

In this study, we have used contemporary research methods to systematically assess the effects of ongoing exposure to security threats. CTS refers to situations of exposure to ongoing threats of individuals, families, and whole communities. Hostility outbursts in these situations are targeted blindly at specific regions making the whole area unsafe for years at a time. This influences individuals’ life style, behaviors and management of daily routine. Thus, some items that are central to the diagnosis of PTSD (i.e., avoidance) are essential to routine of safe behavior, not of safety behavior. Referring to criteria D, there is no reference to betrayal nor to exhaustion which are both central to CTS. We therefore suggest that CTS is a focused measure of long-term exposure to threat resulting in *exhaustion and detachment; rage and betrayal*; *fear and helplessness*, which are not alien to PTSD but are specific to the case we studied.

The findings of this study add to the evolving understanding of the effects of ongoing traumatic stress, suggesting that some stress symptoms that emerge due to ongoing exposure to security threat are distinct from PTSD as currently defined. Future research should confirm the 3-factor structure measured by the CTSR scale and the aspects of threat and psychosocial resources associated with each of the three factors.

To the best of our knowledge, this scale is the first to systematically measure the effects of ongoing traumatic stress. The CTSR offers clinicians a more specific understanding, as well as a reference to the manifestation of PTSD in such circumstances enabling them to identify those in need of intervention, quantitatively assess the effectiveness of treatment, stages in recovery, and the relative efficacy of different interventions. Ideally, this will inform future research leading to better understanding of how the effects of trauma differ in kind and magnitude between acute and ongoing, persistent trauma.

## Supporting information

S1 Table Distance from the Gaza Strip and time to secure shelter. (PDF)

S2 Table Descriptive statistics by exposure to continuous security threat. (PDF)

S3 Table EFA results on 25 ongoing exposure to threat items. (PDF)

S4 Table CTSR predictors: Results of the final adjusted regression model. (PDF)

S1 Fig Scree test plot for exploratory factor analysis (EFA) of the CTSR scale. (TIF)

S2 Fig Correlation between the level of distress caused by CTSR symptoms and total CTSR scale scores. (TIF)

S3 Fig Correlation between the level of impaired functioning caused by CTSR symptoms and total CTSR scale scores. (TIF)

S4 Fig Correlation between total CTSR scale scores and distance from the Gaza border among CTS and non-CTS participants. (TIF)

S1 File The Continuous Traumatic Stress Response (CTSR) scale. (PDF)
