# Correction: Development and validation of the Continuous Traumatic Stress Response scale (CTSR) among adults exposed to ongoing security threats

**DOI:** 10.1371/journal.pone.0253529

**Published:** 2021-06-15

**Authors:** The PLOS ONE Staff

## Notice of republication

An incorrect version of Fig 1 was published in the original version of this article. The publisher apologizes for the error. This article was republished on June 7, 2021 to correct for this error. Please download this article again to view the correct version.
