# Supplementary material for: Development and validation of the Continuous Traumatic Stress Response scale (CTSR) among adults exposed to ongoing security threats
Source: PLoS One. 2021 May 27;16(5):e0251724. doi: 10.1371/journal.pone.0251724 (PMC8158953; doi:10.1371/journal.pone.0251724)
Supplement: S1 Table — (PDF) [file pone.0251724.s001.pdf]

S1 Table. Distance from the Gaza Strip and time to secure shelter

Strip	Distance from the Gaza Strip (km)	Time to secure shelter (sec)
1	10	15
2	20	30
3	30	45
4	40	60
5	50	75
6	60	90

Please refer to: https://image.slidesharecdn.com/mysummervacation-110402192554-phpapp02/95/shared-future-2010-idf-homefront-command-6-728.jpg?cb=1301773572 for a map presenting time to secure shelter throughout Israel.
