# Supplementary material for: Development and validation of the Continuous Traumatic Stress Response scale (CTSR) among adults exposed to ongoing security threats
Source: PLoS One. 2021 May 27;16(5):e0251724. doi: 10.1371/journal.pone.0251724 (PMC8158953; doi:10.1371/journal.pone.0251724)
Supplement: S2 Table — (PDF) [file pone.0251724.s002.pdf]

S2 Table. Descriptive statistics by exposure to continuous security threat

**Demographics characteristic**	**All (N=313)**	**CTS (N=138)**	**Non-CTS (N=175)**	**Test statistic**	**P**
Age, mean (SD)	41.1(13.0)	42.1(14.3)	39.7 (12.1)	t=-2.41, df=299	0.010
Female	189(60.4%)	96(69.6%)	93(53.1%)	χ^2=8.94, df=1	0.001
Married or cohabitating	231(73.8%)	93(67.4%)	138(78.8%)	χ^2=5.10, df=1	0.045
Above average income	104(33.3%)	36(26.1%)	68(38.8%)	χ^2=5.79, df=1	0.024
Non-religious	184(59.1%)	92(66.7%)	92(52.6%)	χ^2=7.54, df=1	0.006
Academic education	215(68.7%)	90(65.2%)	125(71.4%)	χ^2=1.47, df=1	0.220
Israeli*	257(82.1%)	103(74.6%)	154(88.0%)	χ^2=10.91, df=1	0.001
Urban	152(48.6%)	52(37.7%)	100(57.1%)	χ^2=9.73, df=1	0.001
**Trauma related characteristics**					
Prior exposure to trauma	109(34.8%)	44(31.9%)	65(37.1%)	χ^2=0.96, df=1	0.350
PTSD symptoms**	93(29.7%)	37(26.8%)	56(32.0%)	χ^2=1.86, df=1	0.200

CTSR symptoms	140(45.2%)	67(48.6%)	73(42.4%)	χ^2=1.15, df=1	0.280
Fear of events, mean (SD)	2.58(1.16)	3.04(1.15)	2.21(1.02)	t=5.42, df=306	<0.001
Likelihood of events, mean (SD)	2.49(0.77)	2.85(0.75)	2.21(0.66)	t=3.19, df=308	<0.001

*Born in Israel or immigrated before 1990 **Symptoms only, regardless of 'exposure to a traumatic event' as defined by Criteria A
