# Supplementary material for: Development and validation of the Continuous Traumatic Stress Response scale (CTSR) among adults exposed to ongoing security threats
Source: PLoS One. 2021 May 27;16(5):e0251724. doi: 10.1371/journal.pone.0251724 (PMC8158953; doi:10.1371/journal.pone.0251724)
Supplement: S3 Table — (PDF) [file pone.0251724.s003.pdf]

**S3 Table. EFA results on 25 ongoing exposure to threat items (n=113)**

| | Item* | Factor loadings | | |
|---|---|---|---|---|
| | | *1* | *2* | *3* |
| 1 | I feel unmotivated | **.85** | | |
| 2 | I feel mentally exhausted | **.72** | | |
| 7 | I feel that no one understands me | **.70** | | |
| 6 | I find it hard to trust the people around me | **.68** | | |
| 4 | I feel that my life has no meaning | **.51** | | |
| 9 | I have episodes of rage | | **.86** | |
| 5 | I have difficulty controlling my emotions | | **.73** | |
| 10 | I feel betrayed | | **66** | |
| 3 | I feel that my life is in danger | | | **.74** |
| 11 | I feel that I cannot protect the people who depend on me | | | **.68** |
| 8 | I have intense feelings of fear or horror | | | **.43** |
| | Eigenvalues | 5.31 | 1.38 | 1.20 |
| | % of variance | 25.46 | 21.49 | 14.67 |
| | Cronbach's alpha | 0.864 | 0.820 | 0.743 |
| | Factor mean (SD) | 0.55(0.65) | 0.42(0.63) | 0.34(0.53) |
| | Range | 0-2.80 | 0-3.00 | 0-3.00 |

*The English phrases presented in this table are the translation of the original Hebrew phrases
1- *Exhaustion and detachment*; 2 - *Rage and betrayal*; 3 - *Fear and helplessness*
