# Supplementary material for: Development and validation of the Continuous Traumatic Stress Response scale (CTSR) among adults exposed to ongoing security threats
Source: PLoS One. 2021 May 27;16(5):e0251724. doi: 10.1371/journal.pone.0251724 (PMC8158953; doi:10.1371/journal.pone.0251724)
Supplement: S4 Table — (PDF) [file pone.0251724.s004.pdf]

S4 Table. CTSR predictors: Results of the final adjusted regression model

Predictor	High CTSR scores			
	AOR (SE)	P value	95% CI	-2LL*
**Step 1**				365.11
Age	0.96(0.01)	0.003	0.94-0.99	
Gender				
Male	1.00			
Female	1.81(0.28)	0.03	1.05-3.14	
Religiosity				
Secular	1.00			
Traditional	1.39(0.36)	0.53	0.69-2.57	
Religious	0.88(0.34)	0.66	0.45-1.69	
Family status				
Married or cohabitating	1.00			
Single	1.76(0.39)	0.14	0.82-3.79	
Divorced or widow	0.81(0.52)	0.67	0.30-2.18	
Income				
Average	1.00			
Below average	1.22(0.33)	0.54	0.64-2.33	
Above average	0.78(0.33)	0.46	0.41-1.50	
Community type				
Rural	1.00			
Urban	1.04(0.29)	0.89	0.59-1.82	
Past exposure to trauma				
No	1.00			
Yes	1.71(0.28)	0.055	0.99-2.95	
Access to shelter				
No	1.00			
Yes	0.80(0.40)	0.59	0.37-1.76	
Other safe area	0.65(0.47)	0.35	0.26-1.62	
Physical disability				
No	1.00			
Yes	1.51(0.43)	0.33	0.66-3.46	
**Step 2**				357.03
Likelihood of future events	1.11(0.04)	0.007	1.03-1.20	
**Step 3**				356.90
CTS				
Non-CTS	1.00			
CTS	0.89(0.31)	0.73	0.50-1.65	

*-2 Log likelihood
