# Supplementary figures and images for: Development and validation of the Continuous Traumatic Stress Response scale (CTSR) among adults exposed to ongoing security threats
Source: PLoS One. 2021 May 27;16(5):e0251724. doi: 10.1371/journal.pone.0251724 (PMC8158953; doi:10.1371/journal.pone.0251724)

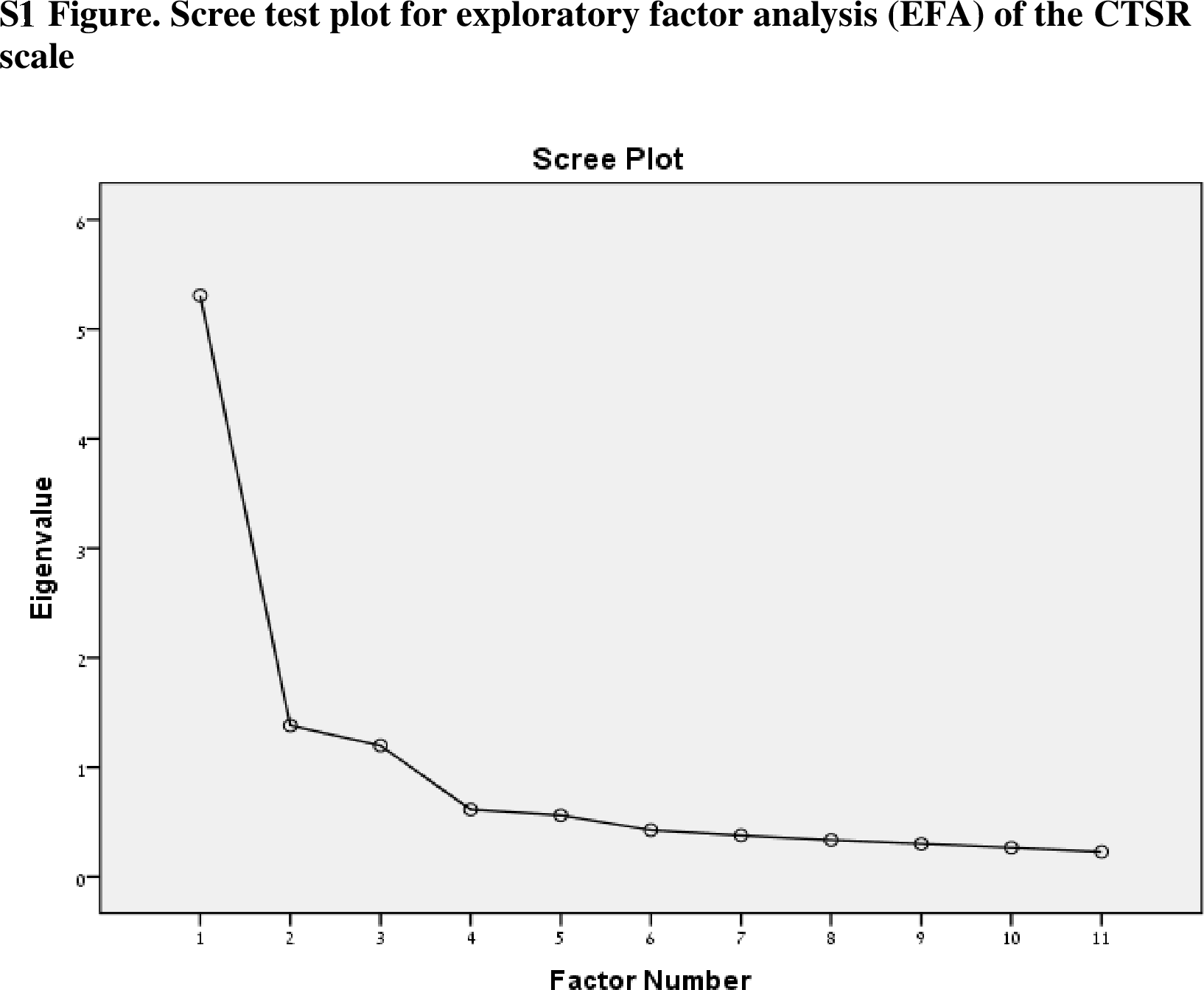

Supplement: S1 Fig — (TIF) [file pone.0251724.s005.tif]

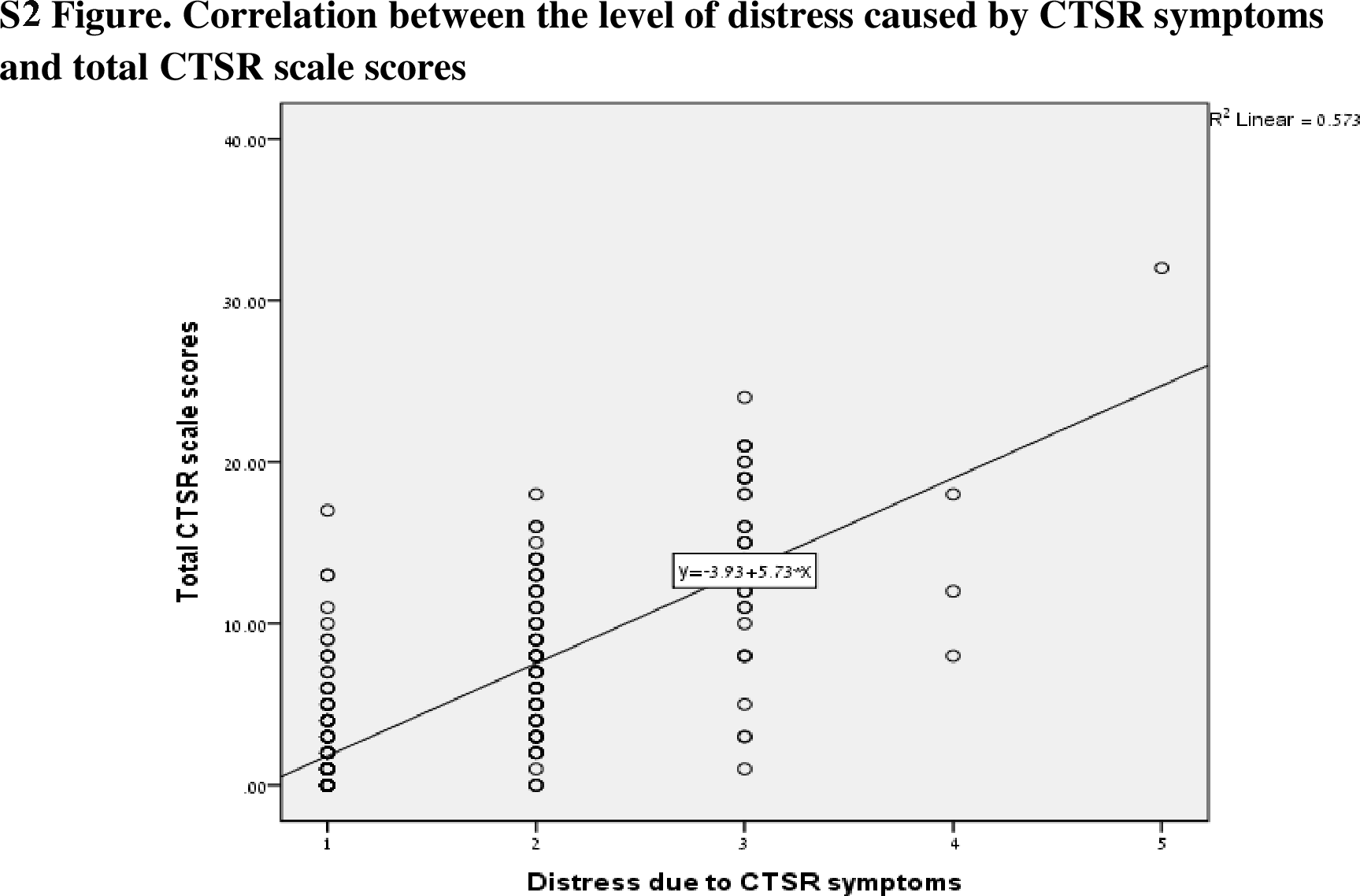

Supplement: S2 Fig — (TIF) [file pone.0251724.s006.tif]

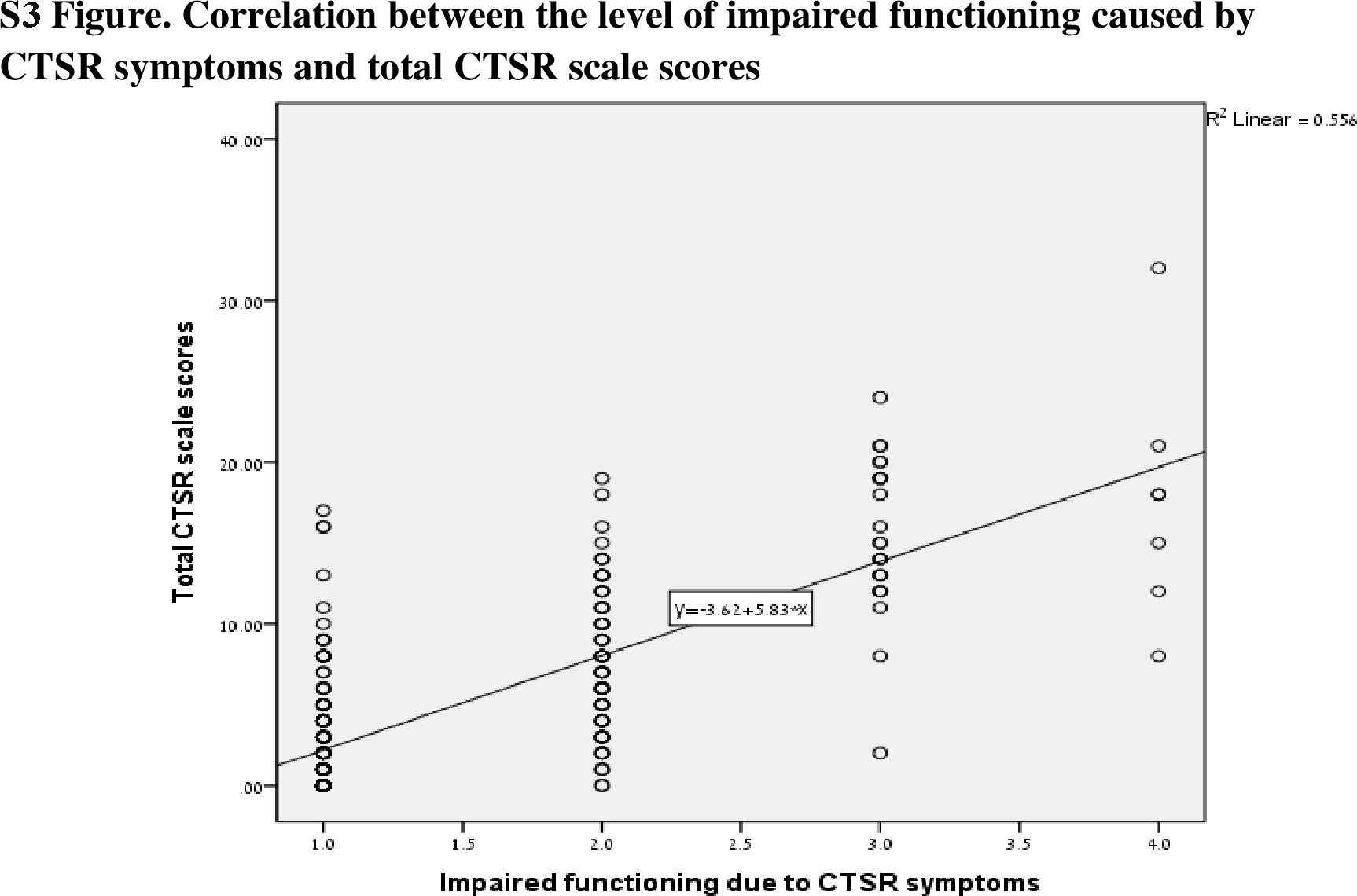

Supplement: S3 Fig — (TIF) [file pone.0251724.s007.tif]

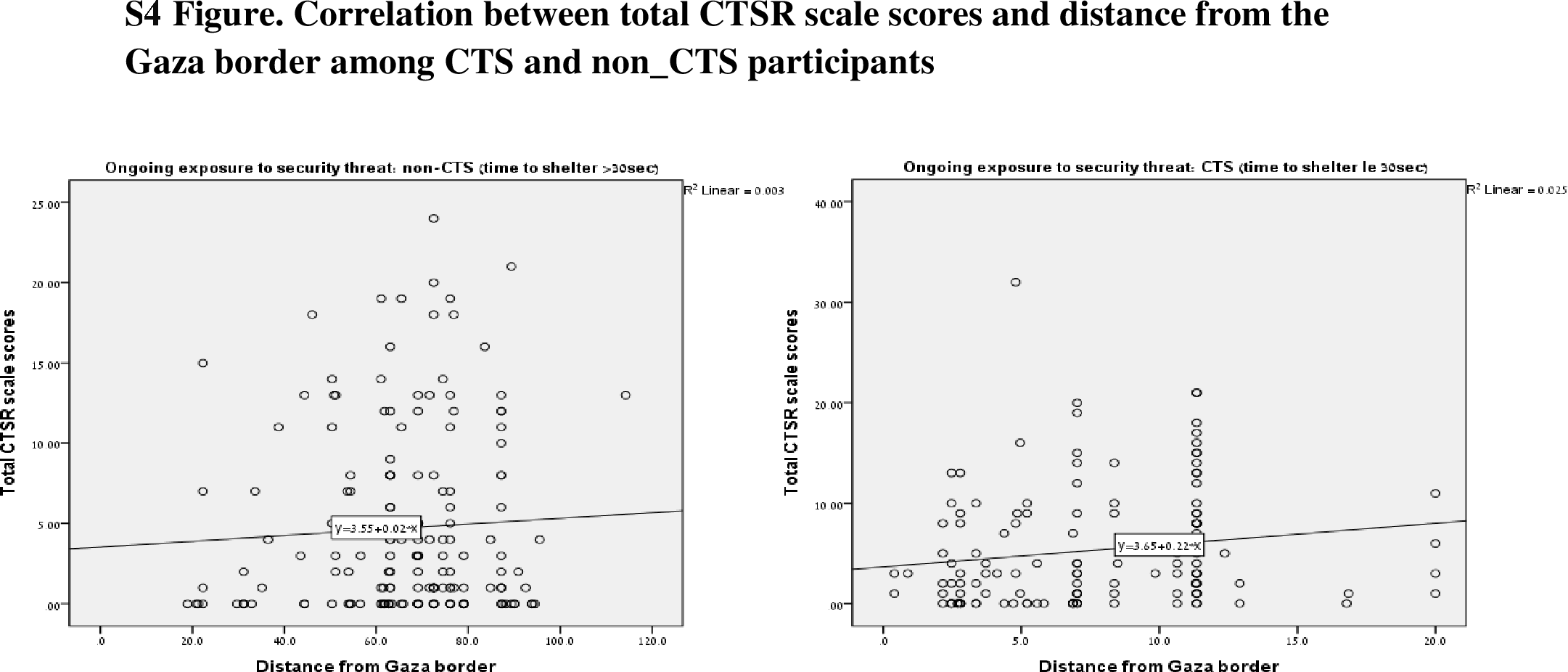

Supplement: S4 Fig — (TIF) [file pone.0251724.s008.tif]
