# Supplementary material for: Development and validation of the Continuous Traumatic Stress Response scale (CTSR) among adults exposed to ongoing security threats
Source: PLoS One. 2021 May 27;16(5):e0251724. doi: 10.1371/journal.pone.0251724 (PMC8158953; doi:10.1371/journal.pone.0251724)
Supplement: S1 File — (PDF) [file pone.0251724.s009.pdf]

S1 File. The Continuous Traumatic Stress Response (CTSR) scale

The following statements describe symptoms that may appear after exposure to severe or stressful events. Please read each statement carefully and mark the answer that best describes your feeling over the past month.

S9A Table. Items included in final scale

Subscale		Item	To what extent does the following statement represents your feeling over the past month			
			Not at all	A little	Very much	Severe
1.	ED	I feel unmotivated	0	1	2	3
2.	ED	I feel mentally exhausted	0	1	2	3
3.	FH	I feel that my life is in danger	0	1	2	3
4.	ED	I feel that my life has no meaning	0	1	2	3
5.	RB	I have difficulty controlling my emotions	0	1	2	3
6.	ED	I find it hard to trust the people around me	0	1	2	3
7.	ED	I feel that no one understands me	0	1	2	3
8.	FH	I have intense feelings of fear or horror	0	1	2	3
9.	RB	I have episodes of rage	0	1	2	3
10.	RB	I feel betrayed	0	1	2	3
11.	FH	I feel that I cannot protect those who depend on me	0	1	2	3

Note. ED = exhaustion and detachment; RB= rage and betrayal; FH= fear and helplessness.

S9B Table. Items not included in final scale

Subscale	Item	To what extent does the following statement represents your feeling over the past month			
		Not at all	A little	Very much	Severe
12.	I feel depressed	0	1	2	3
13.	I hate myself	0	1	2	3
14.	I tend to use medications more than I normally do	0	1	2	3
15.	I have appetite changes	0	1	2	3
16.	I am sick more than usual	0	1	2	3
17.	I feel helpless	0	1	2	3
18.	I feel that I am a victim	0	1	2	3
19.	I feel like I have become a different person	0	1	2	3
20.	I feel changes in atmosphere at home/work	0	1	2	3
21.	I have a tendency to easily get hurt	0	1	2	3
22.	I am experiencing difficulties with my partner	0	1	2	3
23.	I feel as if I was a prisoner	0	1	2	3
24.	I feel frustrated if my needs and desires are not fulfilled immediately	0	1	2	3
25.	I feel that I have no control over the future	0	1	2	3
